# Genetic analyses reveal cryptic introgression in secretive marsh bird populations

**DOI:** 10.1002/ece3.4472

**Published:** 2018-09-05

**Authors:** Stephanie S. Coster, Amy B. Welsh, Gary Costanzo, Sergio R. Harding, James T. Anderson, Susan B. McRae, Todd E. Katzner

**Affiliations:** ^1^ Division of Forestry and Natural Resources West Virginia University Morgantown West Virginia; ^2^ Virginia Department of Game and Inland Fisheries Charles City Virginia; ^3^ Virginia Department of Game and Inland Fisheries Henrico Virginia; ^4^ Department of Biology East Carolina University Greenville North Carolina; ^5^ U.S. Geological Survey Forest and Rangeland Ecosystem Science Center Boise Idaho

**Keywords:** admixture, clapper rail, introgression, king rail, *Rallus crepitans*, *Rallus elegans*

## Abstract

Hybridization is common in bird populations but can be challenging for management, especially if one of the two parent species is of greater conservation concern than the other. King rails (*Rallus elegans*) and clapper rails (*R. crepitans*) are two marsh bird species with similar morphologies, behaviors, and overlapping distributions. The two species are found along a salinity gradient with the king rail in freshwater marshes and the clapper in estuarine marshes. However, this separation is not absolute; they are occasionally sympatric, and there are reports of interbreeding. In Virginia, USA, both king and clapper rails are identified by the state as Species of Greater Conservation Need, although clappers are thought to be more abundant and king rails have a higher priority ranking. We used a mitochondrial DNA marker and 13 diagnostic nuclear single nucleotide polymorphisms (SNPs) to identify species, classify the degree of introgression, and explore the evolutionary history of introgression in two putative clapper rail focal populations along a salinity gradient in coastal Virginia. Genetic analyses revealed cryptic introgression with site‐specific rates of admixture. We identified a pattern of introgression where clapper rail alleles predominate in brackish marshes. These results suggest clapper rails may be displacing king rails in Virginia coastal waterways, most likely as a result of ecological selection. As introgression can result in various outcomes from outbreeding depression to local adaptation, continued monitoring of these populations would allow further exploration of hybrid fitness and inform conservation management.

## 1 INTRODUCTION

Hybridization is the result of a breakdown in interspecific mating barriers, and can result in the genetic introgression of two species. Historically, biologists thought hybridization was maladaptive and irrelevant to speciation (Mayr, 1942, 1963). However, the high frequency of hybridization in natural populations (Mallet, 2005) points to the relevance of such events in influencing evolutionary processes. Among bird species, hybridization is relatively common but infrequent, occurring at least occasionally in one of every ten species (Grant & Grant, 1992). Hybridization among taxa can present a challenge to conservation biologists and managers, especially when a rare species crosses with a common species (Allendorf, Leary, Spruell, & Wenburg, 2001). Such hybridization has been responsible for local extinctions and the breakdown of pure species into predominantly hybrid populations of plants such as the welted thistle (*Carduus acanthoides*) and Catalina mahogany (*Cerocarpus traskaie*) (see Levin, Francisco‐Ortega, & Jansen, 1996 for review), and animals such as red wolves (*Canis rufus*), European mink (*Mustela lutreola*), and New Zealand gray duck (*Anas superciliosa*) to name a few (see Rhymer & Simberloff, 1996 for review and other examples).

Studying hybrid zones can reveal the dynamic relationship between reproductive introgression and isolation and illuminate the nature of species boundaries (Wu, 2001). It also can be useful to study hybrid zones to better understand the population history. For example, hybridization can occur as the result of incomplete differentiation of two lineages or by secondary contact of previously separated populations (Edwards, Soltis, & Soltis, 2008; McGuire et al., 2007). Characterizing the genotypic distribution of a hybrid zone can also elucidate the mechanism of hybridization and suggest the direction of selection on hybrids (Harrison & Bogdanowicz, 1997; Jiggins & Mallet, 2000). The genotypic distribution of a hybrid zone is a continuum; on one end, a hybrid swarm refers to a unimodal distribution where hybrid genotypes predominate, and at the other end is a bimodal distribution where parental forms predominate with few hybrid intermediates (Harrison & Bogdanowicz, 1997; Rubidge & Taylor, 2004).

King (*Rallus elegans*) and clapper rails (*R. crepitans*) are examples of taxa that hybridize (Maley, 2012; Meanley, 1985; Olson, 1997). The two species are difficult to distinguish in their pure forms, as both are secretive and their distributions, diets, calls, and morphometrics overlap (Eddleman & Conway, 1994; Meanley, 1969, 1985; Perkins, King, Travis, & Linscombe, 2009; Stiffler et al., 2017). They are generally distinguished by size and plumage color, with king rails slightly larger (Meanley, 1985) and more deeply rust‐colored than clapper rails (Taylor & van Perlo, 1998). A phylogenetic analysis using mitochondrial DNA suggests they are sister species with discrete populations (Maley & Brumfield, 2013).

Evidence also suggests that king and clapper rail lineages diverged along ecological lines. King rails are found in a diverse range of habitats from freshwater marshes, including rice fields, shrub swamps, and emergent marshes, to coastal brackish marshes (Meanley, 1985; Olson, 1997; Perkins, King, & Linscombe, 2010). Clapper rails are found exclusively in brackish and salt marshes, and are thought to competitively exclude king rails when both are present in sufficient numbers (Eddleman & Conway, 1994; Meanley, 1985; Olson, 1997).

**Figure 1:**
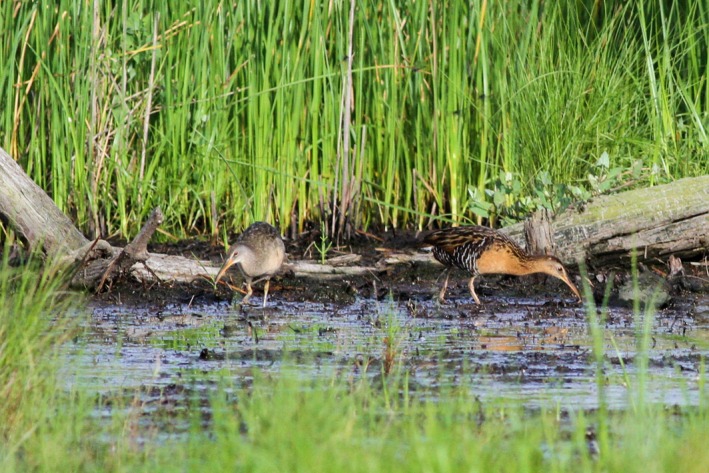
A clapper rail (left) and king rail (right) mated pair. Photo taken by Robert Ostrowski at Truitts Landing in Worcester County, MD

In spite of potential exclusion, both king and clapper rails have been observed in the same breeding grounds (sometimes as mated pairs, see Figure 1) in Delaware, Maryland, New York, Georgia, Alabama, Virginia, and Louisiana (Maley, 2012; Meanley, 1969, 1985). The two species appear to hybridize readily, but only in areas of intermediate salinity or where freshwater and saltwater marshes are found in close proximity (Maley, 2012; Meanley, 1969; Olson, 1997). This spatial arrangement can occur naturally or as the result of anthropogenic manipulation such as the creation of dikes and ditches for impoundment or infrastructure.

The hybridization of king and clapper rails presents management challenges as their conservation status differs. King rails have declined precipitously across much of their range in the last 40 years (Cooper, 2008; Eddleman, Knopf, Meanley, Reid, & Zembal, 1988) and are listed globally as near threatened (IUCN [Link]). King rails are federally endangered in Canada. In the United States, they are locally classified as a Species of Greatest Conservation Need in 30 state Wildlife Action Plans and are categorized as threatened or endangered in a number of states (Cooper, 2008). In contrast, clapper rails are abundant, listed as least concern globally (IUCN [Link]) and appear as a low‐ranked Species of Greatest Conservation Need on only a few state Wildlife Action Plans. Both species are considered game species and are hunted in parts of their range (Raftovich, Chandler, & Wilkins, 2015).

Because of the morphological and behavioral similarities, the range overlap, and the hybridization potential, it can aid management to identify species and evaluate introgression in rail populations that inhabit brackish marshes. The purpose of this study was to use genetic tools to identify the species of rails present at two marshes along a salinity gradient in Virginia and to explore potential introgression within this population. Our specific objectives were to: (a) identify individuals to species using mitochondrial and nuclear genetic markers, and (b) characterize genotypic distribution and examine patterns of introgression.

## 2 MATERIALS AND METHODS

### 2.1 Sampling and DNA extraction

**Figure 2:**
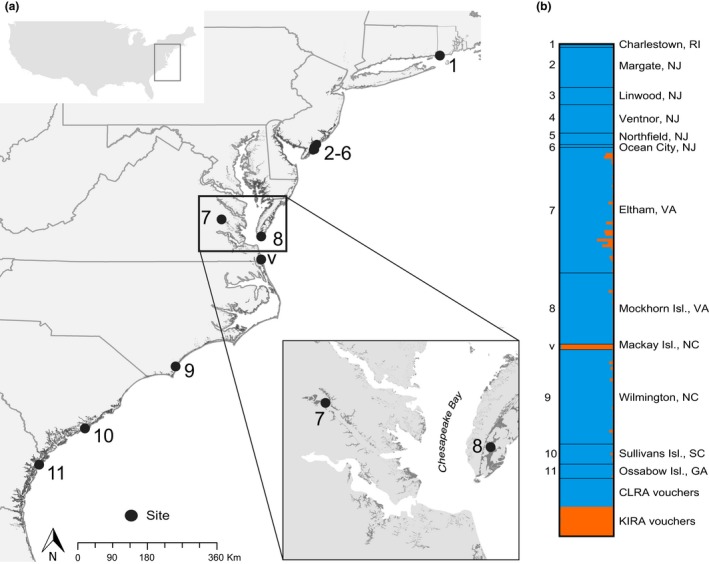
Panel (a) shows the location of sites sampled along the Atlantic coast of the United States (see Table 1) and includes a close up of the two focal areas in Virginia situated along a salinity gradient. Panel (b) is a bar plot of genetic clusters from STRUCTURE for 172 individuals genotyped at 13 diagnostic SNPs. Sites are separated by black lines and ordered from top to bottom (north to south) and numbers correspond to map. Each line shows individual membership to two genetic clusters; blue represents clapper rail’s (CLRA) genetic signature and orange represents king rail’s (KIRA) genetic signature

We concentrated our sampling efforts across a salinity gradient in coastal Virginia. We collected rail samples from two focal areas. The first was Eltham Marsh, a brackish tidal wetland located along the Pamunkey River near West Point, VA and the second was Mockhorn Island Wildlife Management Area, a seaside tidal marsh island off the Eastern Shore of Virginia (Figure 2). We acquired and froze samples from Mockhorn Island (*N* = 25) through hunter harvest in September 2013 or 2014. We thawed the carcasses and used a small amount (<5 mm^2^) of breast tissue in DNA extraction. At Eltham Marsh, we used a thermal imaging infrared camera (Raytheon Thermal‐Eye 250D; Raytheon Company, Waltham, MA) at night to locate rails from an airboat and then a dip net to capture them (Mills et al., 2011) in October 2013 and August–October 2014. We plucked several body or covert feathers from each of 45 individuals and when possible, we also plucked a growing body feather.

Because hybrid analysis requires nonadmixed or pure populations for genotype comparison, we analyzed other hunter‐harvested clapper rail samples collected at saline sites along the Atlantic coast in September or October 2014 to test admixture proportions and identify pure populations (*N* = 88; Table 1; Figure 2). We obtained these samples from New Jersey, North Carolina, South Carolina, and Georgia via the webless migratory game bird “wing‐bee” program administered by the US Fish and Wildlife Service. This event is an annual survey for monitoring purposes where hunters submit clipped wings from harvested birds. We also acquired a sample of breast tissue suspended in 95% EtOH from a single road‐killed clapper rail sample from Charlestown, Rhode Island. For king rail species verification, we obtained two king rail DNA samples captured on a freshwater marsh on Mackay Island, North Carolina. In addition, we obtained mitochondrial sequences from GenBank (king rail accession no. KP081581‐90; clapper rail accession no. KP081591‐600) and nuclear genotypes were shared by J. Maley and R. Brumfield of Louisiana State University.

**Table 1:** Sampling location, site number (corresponding to Figure 2), number of individuals sampled (*N*) including the number of mitochondrial (*N*_mt_) and nuclear DNA samples (*N*_nuc_), the source of DNA and information about voucher specimens (clapper rail–CLRA; king rail—KIRA) for rail populations sampled along the North American Atlantic coast

Sampling location	Site	*N*	*N*_mt_	*N*_nuc_	DNA source
Charlestown, RI	1	1	0	1	Feathers^a^
Margate, NJ	2	20	18	14	Feathers^a^
Linwood, NJ	3	10	10	6	Feathers^a^
Ventnor, NJ	4	15	11	10	Feathers^a^
Northfield, NJ	5	5	5	4	Feathers^a^
Ocean City, NJ	6	1	1	1	Feathers^a^
Eltham Marsh, VA	7	46	45	44	Feathers, tissue, blood
Mockhorn Island, VA	8	25	20	25	Tissue
Wilmington, NC	9	35	35	33	Feathers^a^
Sullivan's Island, SC	10	9	9	7	Feathers^a^
Ossabaw Island, GA	11	7	4	5	Feathers^a^
*KIRA vouchers:* LA	—	—	10	—	GenBank KP081581–90
LA	—	—	—	10	J. Maley (unpubl. data)
Mackay Island, NC	v	2	2	2	Blood
*CLRA vouchers:* LA	—	—	10	—	GenBank KP081591–600
LA	—	—	—	10	J. Maley (unpubl. data)

a Feathers sourced from the USFWS webless migratory game bird wing‐bee.

As DNA was obtained from multiple sources we used a variety of methods for preserving samples and extracting DNA. Feathers were preserved in a dry envelope or in lysis buffer (100 mM Tris‐HCl pH 8, 100 mM EDTA, 10 mM NaCl, 2% SDS). Blood was obtained from the tarsus vein (30–50 μl) and blotted on Whatman filter cards. From each desiccated wing, we plucked a mix of 5–8 primary or covert feathers for DNA extraction. All samples were stored at room temperature. We extracted DNA from blood and tissue samples with a Promega Wizard Genomic DNA Purification kit (Madison, WI) following the manufacturer's protocols. Feather samples were extracted using this kit with the modification of adding 20 μl of 20 mg/ml dithiothreitol (DTT) to the lysis buffer.

### 2.2 Species identification

#### 2.2.1 Mitochondrial sequences

We sequenced mitochondrial DNA for the protein‐coding NADH subunit 2 (ND2) using external primers RallusND2F and RallusND2R (Maley & Brumfield, 2013). The polymerase chain reaction (PCR) contained ~20 ng eluted DNA, 5X GoTaq^®^ Flexi buffer, 0.5 μM of each primer, 1.0 mM MgCl_2_, 0.1 mg/ml bovine serum albumen (BSA), 0.1 μM dNTPs, and 1 unit of GoTaq^®^ DNA Polymerase (Promega, Madison, WI), in 10 μl total reaction volume. We ran a PCR procedure of 2 min at 94°C, then 35 cycles of 94°C for 30 s, 51°C for 30 s, and 72°C for 1 min, then 72°C for 10 min. We purified PCR products using a QIAquick 96 PCR purification (Qiagen, Valencia, CA) kit following the manufacturer's protocol and eluted with 50 μl of water. We then ran cycle‐sequencing reactions using the forward primers with BigDye™ Terminator v3.1 Cycle Sequencing Kit (ThermoFisher) on an ABI 3130XL Genetic Analyzer (Applied Biosystems, Foster City, CA) following the manufacturer's protocols. For sequence verification under a 15 bp “dye‐blob”, we also ran all samples with cycle‐sequencing reactions with the GenomeLab DTCS Quick Start Kit on a Beckman Coulter GeXP Genetic Analyzer (Brea, CA) following the manufacturer's protocol.

Mitochondrial sequences were viewed and edited for quality using Finch TV 1.5.0 (Geospiza Inc, Seattle, WA, USA), and a consensus sequence was created for each individual. We aligned sequences using CLC Sequence Viewer 7 (Qiagen, https://www.qiagenbioinformatics.com/products/clc-sequence-viewer-direct-download/) and identified species by comparing sequences and looking at eight diagnostic polymorphisms in the 620 bp sequence. We identified individuals to species when ≥6 polymorphisms aligned to the voucher specimens in GenBank, as this was the minimum number of diagnostic alleles among the voucher specimens.

#### 2.2.2 Nuclear genotypes

We identified 13 diagnostic single nucleotide polymorphism (SNP) loci that displayed high allele frequency differences between king and clapper rails (frequency difference >0.7, mean = 0.96, *SD* = 0.08; Maley & Brumfield, 2013). Primers for four loci (139, 472, 1166, and 1766) were acquired from Maley (2012). We designed primers for the remaining loci using Primer3 (Koressaar & Remm, 2007; Untergasser et al., 2012) directly from the 454 sequencing reads (Maley & Brumfield, 2013; Supporting Information Appendix Table S1).

We genotyped samples using high‐throughput genotyping following the genotyping‐in‐thousands (GT‐seq) protocol (Campbell, Harmon, & Narum, 2015). This protocol uses i5 and i7 Illumina index primers to uniquely identify each locus and well in a 96‐well plate. We therefore pooled all loci and samples across two plates for sequencing. The only amendment to the published library preparation protocol (Campbell et al., 2015) was that we revised the PCR conditions in the first round of PCR (PCR1) to increase amplification using a touchdown procedure as follows: 95°C for 5 min, followed by 15 cycles of 95°C for 30 s, annealing temperatures starting at 65°C for 90 s then decreasing 1°C per cycle, and 72°C for 30 s for extension. This step was followed by 15 cycles of 95°C for 30 s, 50°C for 90 s, and 72°C for 30 min with a final extension at 68°C for 12 min. We ran sequencing reactions using MiSeq Reagent Kit v2 Nano on an Illumina MiSeq with paired‐end reads and a read length truncated at 200 bp.

Sequencing data were concatenated into a single fastq file, and the index sequences were used to split by plate and sample. We then used the bioinformatics pipeline (v1) from Campbell et al. (2015) to run perl scripts and genotypes were determined by the ratio of allele 1 to allele 2 probes. Loci with total read counts less than 10× were not scored, and those samples not scored at ≥4 loci were dropped from the analysis. To document, how informative the diagnostic SNP markers were in our samples, we calculated the allele frequencies in both species across the populations classified as “pure”.

We used the Bayesian clustering approach of STRUCTURE 2.3.4 (Pritchard, Stephens, & Donnelly, 2000) to confirm that the diagnostic nuclear loci clearly differentiated the two species in our sampled populations. We first investigated the most parsimonious number of subpopulations (*K*) by conducting five runs for each value of *K* = 1−3; each run consisted of 50,000 burn‐ins followed by 100,000 iterations modeled with admixture and correlated allele frequencies. We tested this range of *K* with the understanding that if *K* = 1 had the highest probability, this suggests the loci cannot clearly differentiate the species and if the highest probability was *K* = 3 then the markers may be too sensitive to differentiate species. We confirmed the optimal number of subpopulations by reviewing the bar plot for coherence, averaging the likelihood over all runs for each *K* and evaluating the peak probability using the software STRUCTURE HARVESTER (Earl & vonHoldt, 2012) following the evaluation method of Pritchard et al. (2000) (Supporting Information Appendix Figure S1).

### 2.3 Classifying introgression

Although the program STRUCTURE produces as output a *Q* value, or the proportion of each individual's genotype belonging to each species, this value does not allow classification of genotypes into hybrid classes based on generation or backcrossing. To identify hybrid classes and quantify introgression, we used the R package *introgress* (Gompert & Buerkle, 2009, 2010). This package requires reference files of pure and admixed populations. To create these reference files, we used the STRUCTURE model with *K* = 2, and combined sampling sites into three groups (pure king, pure clapper, or admixed) based on the *Q* value. Sampling sites where all individuals had a *Q* value ≥0.98 for one species were classified as pure king or clapper populations. We used this conservative cutoff value as this was the minimum *Q* value from our voucher specimens. If one or more individual at a sampling site had a *Q* value between 0.50 and 0.98, we considered the sampling site admixed.

To classify individuals as pure, recent hybrids (first or second generation) or past hybrids (backcrossed), we evaluated two metrics, the hybrid index and interspecific heterozygosity. The hybrid index, or admixture coefficient, is the proportion of alleles inherited from king rails (0 = clapper rail, 1 = king rail). Interspecific heterozygosity clarifies the timing of the hybridization event, because with fixed alleles, first‐generation hybrids are 100% heterozygous with declining heterozygosity over generations. Interspecific heterozygosity ranges from 0 to 1 (0 = all homozygous genotypes, 1 = all heterozygous genotypes). Using the combination of these two metrics, we assigned individuals into genotypic classes. We conservatively classified individuals as first or second‐generation hybrids (*F*_1_, *F*_2_) if they had an intermediate hybrid index (0.25–0.75) and high heterozygosity (>0.3) (see Milne & Abbott, 2008; Walsh, Shriver, Olsen, O'Brien, & Kovach, 2015). For backcrossed and pure classifications, if an individual had a hybrid index between 0.05 and 0.25, we classified them as backcrossed and if their hybrid index was <0.05 we classified them as pure.

## 3 RESULTS

We acquired a total of 174 phenotypic clapper rail samples (of which 71 were from Virginia) and two known king rail samples. We successfully extracted mitochondrial DNA from 160 of these and nuclear DNA from 152 of them. We used an additional 20 mitochondrial DNA sequences and 20 nuclear DNA sequences from king and clapper rails sampled in Louisiana as reference sequences (Table 1).

### 3.1 Species identification

#### 3.1.1 Mitochondrial sequences

We dropped one sample from the analysis due to a poor quality sequencing read. The known king rail samples from North Carolina and Louisiana were identified as king rails. Among the other samples, 97% were identified as clapper rails (*N* = 168; GenBank accession numbers for mtDNA: MG981763−MG981913). In the focal areas, five samples from live‐captured birds phenotypically identified as clapper rails were classified as king rails based on the mitochondrial DNA analysis (*N* = 4 from Eltham and *N* = 1 from Mockhorn). These five individuals were therefore noted as potential hybrids pending analysis of nuclear DNA. All samples collected from outside the focal area (i.e., from the wing‐bee) were classified as clapper rails from the mtDNA analysis.

#### 3.1.2 Nuclear genotypes

Sequencing of SNP loci produced 5.6 M total reads, with 2.7 M reads per plate. Read counts from individual samples averaged 31,869 (*SD* = 20,403). The average percent of target genotypes per sample collected was 90.2% (*SD* = 20.4%). From the 176 samples, 142 produced genotypes in at least 90% of the targeted loci. We omitted 25 samples from further analyses as they failed at >30% of loci.

### 3.2 Classifying introgression

The STRUCTURE analysis indicated that *K* = 2 and *K* = 3 both had high probability. After reviewing the bar plot, we accepted the smallest *K* value (*K* = 2) that fit the data and was biologically reasonable (following Pritchard, Wen, & Falush, 2010), and we confirmed the SNP loci had sufficient power to differentiate the species. The STRUCTURE analysis corroborated the reference king and clapper rail genotypes from Louisiana and North Carolina were pure and not admixed. All samples from New Jersey were also classified as pure clapper rails. For these pure populations, we found the diagnostic SNP markers had a mean allele frequency difference of 0.87 (Supporting Information Appendix Table S2). Admixed individuals were found in the populations from our two focal areas of Virginia, as well as samples from North Carolina, South Carolina, and Georgia (Figure 2).

**Figure 3:**
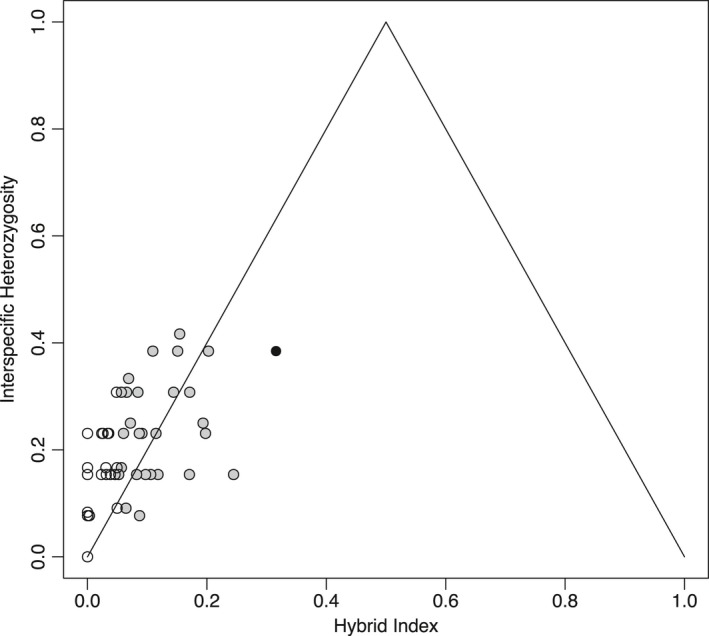
Interspecific heterozygosity (0 = all homozygous genotypes, 1 = all heterozygous genotypes) plotted against hybrid index (0 = clapper rail, 1 = king rail) for 114 individuals sampled from the admixed populations (sites 7−11). The hybrid index is the proportion of alleles derived from the king rail. Individuals were classified as F1/F2 hybrids (black), backcrossed (gray), and pure (white)

Investigating hybrid index and heterozygosity from *introgress* further refined admixed classifications into first‐ and second‐generation hybrids (*N* = 1) and backcrossed clapper rails (*N* = 28). The results show an unequal genotypic distribution with a bias toward clapper rails. The genotypic distribution is not typical of a hybrid swarm as no individuals had an intermediate hybrid index (from 0.4 to 0.6) and no individuals had an interspecific heterozygosity >0.5 (Figure 3). The focal areas had the highest percent of admixed individuals of all sampling sites. At Mockhorn, 12% of individuals were classified as backcrossed. At Eltham, one individual was classified as a recent hybrid and 40% were classified as backcrossed (Table 2).

**Table 2:** Average hybrid index, average interspecific heterozygosity, and introgression class summary of the two focal sites in Virginia and other sites sampled along the Atlantic coast in the USA with admixed individuals grouped by state

Site	Sample size	Ave. hybrid index (*SD*)	Ave. int. het	No. 1/2 gen hybrid	No. backcrossed	Percent backcrossed (%)
Eltham (VA)	44	0.064 (0.08)	0.19	1	17	38.6
Mockhorn (VA)	25	0.016 (0.03)	0.15	0	3	12.0
NC	33	0.017 (0.04)	0.11	0	5	15.2
SC	7	0.021 (0.03)	0.08	0	2	28.6
GA	5	0.021 (0.04)	0.08	0	1	20.0

Combined genotypes from nuclear and mitochondrial markers showed a pattern where the majority of individuals exhibiting introgression had clapper rail mitochondrial DNA and mixed nuclear DNA (*N* = 19). A few individuals classified phenotypically as clapper rails had king rail mitochondrial DNA and either mixed (*N* = 1) or pure clapper rail nuclear DNA (*N* = 4).

## 4 DISCUSSION

This study shows the utility of both nuclear and mtDNA to explore species identity and genotypic distribution in a hybrid zone. The focal populations show evidence of king rail alleles in these clapper rail populations. Our results indicate that the majority of introgression should be classified as backcrossed, suggesting that hybrids are viable, but that in these brackish marshes there is asymmetrical admixture where clapper rail alleles predominate both in the mitochondrial and nuclear markers.

In both focal populations we evaluated, parental clapper rail genotypes predominated. As there are no pure king rail genotypes in the focal populations, the results are displaying one‐half of a bimodal distribution where parental genotypes outnumber hybrid genotypes. The paucity of first‐ and second‐generation hybrids suggests a higher fitness for clapper rails in these populations. Genetic and physical evidence indicate the two species have diverged relative to osmoregulation, with clapper rails having larger salt glands and the ability to excrete salt more quickly at higher concentrations (Conway, Hughes, & Moldenhauer, 1988; Maley, 2012).

While there is evidence that the pattern of introgression we observed resulted from ecological selection, there are several possible explanations for how these two species came to share alleles. For example, one option is that king and clapper rails have not yet fully diverged and continue to share ancestral alleles (i.e., incomplete lineage sorting). Alternatively, it is possible that gene flow between these two previously isolated species has occurred due to recent mixing (i.e., secondary contact). Based on the first‐hand accounts of inter‐species matings (Eddleman & Conway, 1994; Maley & Brumfield, 2013; Meanley, 1969, 1985; Perkins et al., 2009), we believe the scenario of secondary contact is more likely. This supports Olson's (1997) hypothesis concerning the evolutionary history of king and clapper rails based on morphology, distribution, and paleontology. He suggested that an ancestral king rail lineage populated North America and was adapted to freshwater marshes. King rails at the range periphery then became isolated by high seas in the Caribbean, and subsequently adapted to salt marshes, becoming the clapper rail. The clapper rail lineage recolonized North America during the Wisconsinan glaciation (75,000–11,000 years ago) and spread northward along the Atlantic coast, incompletely displacing the king rail. This proposed evolutionary history is supported by the extensive range of the king rail compared to the more specialized range of the clapper rail, the competitive exclusion demonstrated by clapper rails in salt marshes, and the common occurrence of hybridization between species in marshes of intermediate salinity at some locations where range overlap occurs (Eddleman & Conway, 1994; Meanley, 1969; Olson, 1997). Our study is consistent with this hypothesis.

Other studies of avian hybrid zones have documented asymmetrical introgression where genotypes of one species predominate (Rohwer, Bermingham, & Wood, 2001; Secondi, Faivre, & Bensch, 2006; Taylor et al., 2014; Walsh, Shriver, Olsen, & Kovach, 2016). This pattern is often associated with range expansion or a shifting hybrid zone. However, without tracking populations through time, it can be difficult to identify which species is invading and which species is being displaced. Although early assumptions led to the prediction that invading genotypes move into the receding species, simulations now show that the opposite occurs (Currat, Ruedi, Petit, & Excoffier, 2008). During the early stages of introgression when there are just a few invaders, interbreeding introduces genotypes from the receding species into the invading species. As the size of the invading population increases, both neutral and selected genotypes trickle into the invading species (Currat et al., 2008); this movement has been described as a “wave‐front” model.

When applied to our data, this “wave‐front” model suggests that across our focal areas clapper rails have displaced king rails. We found the focal site with intermediate salinity (Eltham; 0−15 PPT) had higher frequency of introgression than the more saline site (Mockhorn; 14−31 PPT). These introgression levels match a scenario in which clapper rails have progressively invaded inland along the decreasing salinity gradient. In addition to our results, historic data also support the hypothesis of king rail displacement by the clapper rail. A previous call back survey at brackish marshes adjacent to our focal site along the Pamunkey River and conducted approximately a decade before this study, reported visual and auditory responses exclusively from king rails (Paxton & Watts, 2002). That we did not capture a single king rail at the focal sites during this study indicates that they may have been entirely displaced.

Similar patterns of displacement have been reported throughout the Chesapeake Bay region. In Maryland, records suggest king rails were fairly common in tidal wetlands until the 1980s (Blom et al., 1996; Stewart & Robbins, 1958) but declined precipitously by the 1990s (Brinker et al., 2002). This decline coincided with increased abundance and spread of clapper rails (Brinker et al., 2002). In addition, king rail decline and clapper rail invasion have been observed along the James River in Virginia (Bryan Watts, pers. comm.).

In the case of king and clapper rails, environmental factors associated with climate change are most likely facilitating the invasion of clapper rails and displacement of king rails in these brackish marshes. Climate change influences salt marsh vegetation as sea levels rise and increased salinity results in plants with greater tolerance for inundation replacing those less tolerant (Erwin, Sanders, Prosser, & Cahoon, 2006; Jarrell, Kolker, Campbell, & Blum, 2016; Warren & Niering, 1993). The relatively rapid sea level rise that occurred between 1985 and 1995 (Church & White, 2006; Jarrell et al., 2016) corresponds temporally with documented king rail decline and clapper rail expansion (Brinker et al., 2002). In addition, anthropogenic marsh manipulation such as ditching, dredging, flooding, or filling may exacerbate displacement by altering salinity and bringing the two species into contact (e.g., Maley, 2012; Olson, 1997).

Sexual selection associated with female choice may also play a role along the invasion front (e.g., Stein, Uy, & Nürnberger, 2006), leading to hybridization and displacement. Avian characteristics commonly associated with female choice include plumage color (Hill, 1991; Liu, Siefferman, Mays, Steffen, & Hill, 2009; Norris, 1990) and vocal repertoire (Nowicki, Hasselquist, Bensch, & Peters, 2000; Searcy, 1992; Yasukawa, Blank, & Patterson, 1980). These traits are distinct in king and clapper rails, with king rails larger and more rufescent, and exhibiting less call variation (Meanley, 1985). Our mtDNA results suggest that female clappers may be mating with male kings as we found four times more hybrids with clapper rather than king rail maternally inherited mitochondrial DNA. That said, repeated backcrossing to clapper rails could contribute to this pattern.

Following a genic view of speciation (Wu, 2001), an alternative explanation for our results is that we sampled a stable hybrid cline along a salinity gradient and that the phenotypic and genetic skew toward clapper rails in brackish marshes is due to their higher tolerance of osmoregulatory stress. In this scenario, neutral DNA from king rails percolate into the clapper genome through limited hybridization and diffuse through the porous species boundary because these markers are invisible to selection. However, when taking into consideration the documented salinity changes (Church & White, 2006; Jarrell et al., 2016), species’ occupancy patterns at other brackish marshes in the Chesapeake Bay watershed (Blom et al., 1996; Brinker et al., 2002; Stewart & Robbins, 1958), and the “wave‐front” model of hybridization (Currat et al., 2008), we believe the displacement scenario has greater support. Similar patterns of displacement have been documented in populations of warblers (Rohwer et al., 2001; Secondi et al., 2006) and marsh passerines (Walsh et al., 2016). In both of these examples, displacement is linked in part to environmental change, with forest regeneration causing the blue‐winged warbler (*Vermivora cyanoptera*) (Gill, 1980) to supplant the golden‐winged warbler (*V. chrysoptera*) (Gill, 1980 *)* and salt marsh degradation causing the Nelson's sparrow (*Ammodramus nelsoni*) to supplant the Saltmarsh sparrow (*A*. *caudacutus*; Walsh et al., 2017).

Identifying hybrids in the king‐clapper rail complex using phenotype alone is unreliable and genetic methods are needed to verify admixture. All individuals found in our focal sites in Virginia were identified phenotypically in the field as putative clapper rails. Hybrids identified genetically were not distinguishable in the field by phenotype alone. Other studies have illustrated that hybridizing species are sometimes misidentified in the field (e.g., Ford, Selman, & Taylor, 2017). For example, in the golden‐blue‐winged warbler complex some hybrids are indistinguishable from the parental taxa and there is extensive cryptic genetic variation that occurs before notable phenotypic variation (Vallender, Robertson, Friesen, & Lovette, 2007). Unfortunately, this complicates attempts to monitor introgression in rails, as these species are elusive and difficult to catch.

This study was focused in a brackish marsh system, and our ability to make inferences on hybrid zones does not extend to freshwater systems where king rails may predominate. At our study site along the Pamunkey River, rail occupancy substantially declines up river (see Stiffler et al., 2017), and we were thus unable to explore introgression along the entire salinity gradient. That said, at the sites we sampled, it is possible that hybridization rates may be higher than those we detected for several reasons. First, hybrids will evade detection if they have poor survival and low fitness and we would be unlikely to detect unfit individuals that die early. Second, we used diagnostic nuclear markers to identify hybrids. These markers are predicted to be under divergent selection and thus exhibit reduced introgression (see Yuri, Jernigan, Brumfield, Bhagabati, & Braun, 2009), and therefore conservatively estimate hybridization.

Genetic introgression such as we observed has implications for management. Introgression can threaten taxonomic integrity by creating a hybrid swarm or can result in reduced fitness or outbreeding depression (Edmands, 2007; Rhymer & Simberloff, 1996). Introgression can also generate novel genotypes that increase fitness or lead to local adaptation (Dowling & Secor, 1997; Rhymer & Simberloff, 1996). Our study suggests that rail populations in brackish marshes with connectivity to freshwater are likely to exhibit some introgression between king and clapper rails. When considering the management implications of introgression in this system, it is important to recognize several relevant points. First, introgression is likely influenced by both natural (e.g., climate change) and anthropogenic (e.g., ditches or diking) factors and management strategies should be selected with this in mind. Second, both species are native and thus have intrinsic conservation value, despite the conservation status of each being different, with king rails currently under greater threat than clapper rails. Third, although patterns of introgression suggest clapper rails are displacing king rails in hybrid zones, clapper rails are not known to invade freshwater marshes, so there should still be habitat available for king rails. Our results therefore suggest that long‐term monitoring may be important to understanding the consequences of climate change on introgression and to explore hybrid fitness in this system.

## CONFLICT OF INTEREST

None declared.

## AUTHOR CONTRIBUTIONS

S.S.C. and A.B.W. conceived and designed the study, with input from T.E.K., J.T.A., S.R.H, and G.C. Funding was obtained by T.E.K., A.B.W., and J.T.A.. G.C. and S.B.M. collected samples with help from S.S.C. and others. S.S.C. conducted the laboratory work, analyzed data, and wrote the manuscript with input from all authors, especially A.B.W., T.E.K. and J.T.A. All authors contributed to revisions.

## DATA ACCESSIBILITY

Mitochondrial sequences (GenBank accession no. MG981763−MG981913). Nuclear SNP genotypes and sampling location are in Dryad Digital Repository: https://doi.org/10.5061/dryad.46cn27g.
