# Supplementary material for: Genetic analyses reveal cryptic introgression in secretive marsh bird populations
Source: Ecol Evol. 2018 Sep 5;8(19):9870–9. doi: 10.1002/ece3.4472 (PMC6202719; doi:10.1002/ece3.4472)
Supplement: Supplementary file 2 [file ECE3-8-9870-s002.docx]

**Appendix Figure 1.** Population clusters as identified using 13 diagnostic SNPs in the program STRUCTURE using the mean estimated logarithm probability of the data.

**Appendix Table 1**. Primers used to amplify 13 diagnostic SNPs for king and clapper rail.

| Primer ID | Forward | Reverse |
| --- | --- | --- |
| 472 | GGT ATG CAG CTC ACC ACA GA | GCA AGA AAG AGG CTG TGT CC |
| 139 | CTG GGA GTG AGG GCA GAG | CCT TGC AAG TTG ATG GAT GA |
| 1766 | GCA GCA AAC TAG GAG CAG TAC A | CTT CAG CCT CCC ATC AAG G |
| 1166 | CCT GTG CTC CTG CTG ATG TA | CAA CCA GAT GTT GCA GGA GA |
| 38 | AAC CTG CTG CTC ATC CTC AT | CCT GAG AGT GGG CTT TGT CT |
| 42 | CAT GTC TAT TCG GGG AAT ACC | GCA GAT GAG GAA GGA GAG GA |
| 50 | AGG TTA GCA GGG AAG CCA GT | GAG TGC TGG GAA GAG TGT CC |
| 54 | GCA GCA CTG CAC ATG AAA AT | TTT GAG CTG CTC TCC TGG TT |
| 329 | TCA GTG TCT CCA AAC CCA CA | CCC TCT TTT CCA CAC TGG AG |
| 431 | AAG CTT GAT GGA AGC TGT CG | CGC TCC CAG TCT CTT TCA AC |
| 437 | CAC GGT CTT CCC AGT CCT AA | TCC TCT CCC AGC TCT GTA GC |
| 565 | TCC ATG GAG GAC GGG ATT AT | TTG GCA GAA CCT CAA ACT CA |
| 866 | ATC TGT TGC TCA GCG AGT CC | TGT TGG TGA AAG AAG CAA GC |

**Appendix Table 2.** Allele frequencies for 13 nuclear SNPs pooled from sampling sites classified as pure king rail (KIRA) and pure clapper rail (CLRA) (see Fig. 1). Clapper rail samples were from the Atlantic coast (sites 2−6 ; N = 39) and include voucher specimens from Louisiana (N = 10). King rail samples were from voucher specimens in North Carolina (site v; N = 2) and Louisiana (N = 10).

|  |  |  |  | Allele frequency difference |
| --- | --- | --- | --- | --- |
|  |  | KIRA | CLRA |  |
| Locus | Allele | (N = 12) | (N = 49) |  |
| 1166 | A | 1.00 | 0.00 | 1.00 |
|  | G | 0.00 | 1.00 |  |
| 139 | G | 1.00 | 0.02 | 0.98 |
|  | T | 0.00 | 0.98 |  |
| 1766 | C | 0.95 | 0.01 | 0.94 |
|  | T | 0.05 | 0.99 |  |
| 329 | A | 0.89 | 0.02 | 0.87 |
|  | G | 0.11 | 0.98 |  |
| 38 | G | 1.00 | 0.00 | 1.00 |
|  | A | 0.00 | 1.00 |  |
| 42 | C | 0.91 | 0.16 | 0.75 |
|  | T | 0.09 | 0.84 |  |
| 431 | T | 0.80 | 0.01 | 0.79 |
|  | G | 0.20 | 0.99 |  |
| 437a | G | 1.00 | 0.38 | 0.62 |
|  | A | 0.00 | 0.62 |  |
| 472a | T | 1.00 | 0.17 | 0.83 |
|  | C | 0.00 | 0.83 |  |
| 50 | G | 0.91 | 0.00 | 0.91 |
|  | A | 0.09 | 1.00 |  |
| 54 | G | 1.00 | 0.05 | 0.95 |
|  | A | 0.00 | 0.95 |  |
| 565a | C | 0.79 | 0.05 | 0.74 |
|  | G | 0.21 | 0.95 |  |
| 866 | G | 0.90 | 0.01 | 0.89 |
|  | T | 0.10 | 0.99 |  |
|  | Mean allele frequency difference | | | 0.87 |
